# A spatial portrait of the human sebaceous gland transcriptional program

**DOI:** 10.1016/j.jbc.2024.107442

**Published:** 2024-06-03

**Authors:** Maria Schmidt, Florian Hansmann, Henry Loeffler-Wirth, Christos C. Zouboulis, Hans Binder, Marlon R. Schneider

**Affiliations:** 1 Interdisciplinary Centre for Bioinformatics (IZBI), University of Leipzig, Leipzig, Germany; 2 Veterinary Faculty, Institute for Veterinary Pathology, University of Leipzig, Leipzig, Germany; 3 Departments of Dermatology, Venereology, Allergology and Immunology, Staedtisches Klinikum Dessau, Brandenburg Medical School Theodor Fontane and Faculty of Health Sciences Brandenburg, Dessau, Germany; 4 Institute of Veterinary Physiology, Veterinary Faculty, University of Leipzig, Leipzig, Germany

**Keywords:** sebaceous gland, skin, spatial transcriptomics, pseudotime, holocrine

## Abstract

Sebaceous glands (SG) and their oily secretion (sebum) are indispensable for maintaining skin structure and function, and their deregulation causes skin disorders including but not limited to acne. Recent studies also indicate that sebum may have important immunomodulatory activities and may influence whole-body energy metabolism. However, the progressive transcriptional changes of sebocytes that lead to sebum production have never been characterized in detail. Here, we exploited the high cellular resolution provided by sebaceous hyperplasia and integrated spatial transcriptomics, pseudo time analysis, RNA velocity, and functional enrichment to map the landscape of sebaceous differentiation. Our results were validated by comparison with published SG transcriptome data and further corroborated by assessing the protein expression pattern of a subset of the transcripts in the public repository Human Protein Atlas. Departing from four sebocyte differentiation stages generated by unsupervised clustering, we demonstrate consecutive modulation of cellular functions associable with specific gene sets, from cell proliferation and oxidative phosphorylation *via* lipid synthesis to cell death. Both validation methods confirmed the biological significance of our results. Our report is complemented by a freely available and browsable online tool. Our data provide the first high-resolution spatial portrait of the SG transcriptional landscape and deliver starting points for experimentally assessing novel candidate molecules for regulating SG homeostasis in health and disease.

Hair follicle-associated sebaceous glands (SG) play a pivotal role in maintaining the skin's health. In a process called holocrine secretion, committed peripheral cells massively engage in lipid synthesis, are dislodged towards the middle of the gland, and eventually rupture, releasing an oily secretion (sebum) that lubricates and protects the integument (1, 2). Sebum has additional important but largely unexplored roles, such as protecting against UVB-induced apoptosis (3), contributing to skin’s innate immune response (4), and modulating whole-body energy metabolism (5). Notably, SG deregulation is key for acne pathogenesis (6), and plays a role in other debilitating skin diseases such as atopic dermatitis and psoriasis (7).

Single-cell transcriptomics has been widely applied to study cellular heterogeneity and lineage pathways during human skin development, homeostasis, and diseases (8, 9). Except for a recent scRNAseq study assessing mouse SG cells (10), SG cells have been largely neglected in such studies, because they are absent in the samples or outside the focus of the study. Notably, a comparison of scRNA datasets revealed that human and mouse SG operates largely distinct differentiation programs (11). Single-cell transcriptomics, however, does not directly provide information on tissue organization and cell-cell interactions, and situ techniques such as multiplex immunohistochemistry or imaging mass cytometry allow only the analysis of a limited number of targets. Spatial transcriptomics (ST), in contrast, allows an understanding of complex cell-cell communication by unifying molecular profiles and spatial information within the native tissue context (12). A discernment of sebocyte differentiation stages was not achieved in recent spatial transcriptomics-based (13) or laser capture microdissection-based (14) assessments of human SGs. Here, we report the first in-depth spatial analysis of the human SG transcriptome and provide a detailed and finely granulated characterization of the SG transcriptional landscape and of the sebaceous differentiation program.

## Results and discussion

### Unsupervised clustering depicts four sebocyte differentiation stages

**Figure 1:**
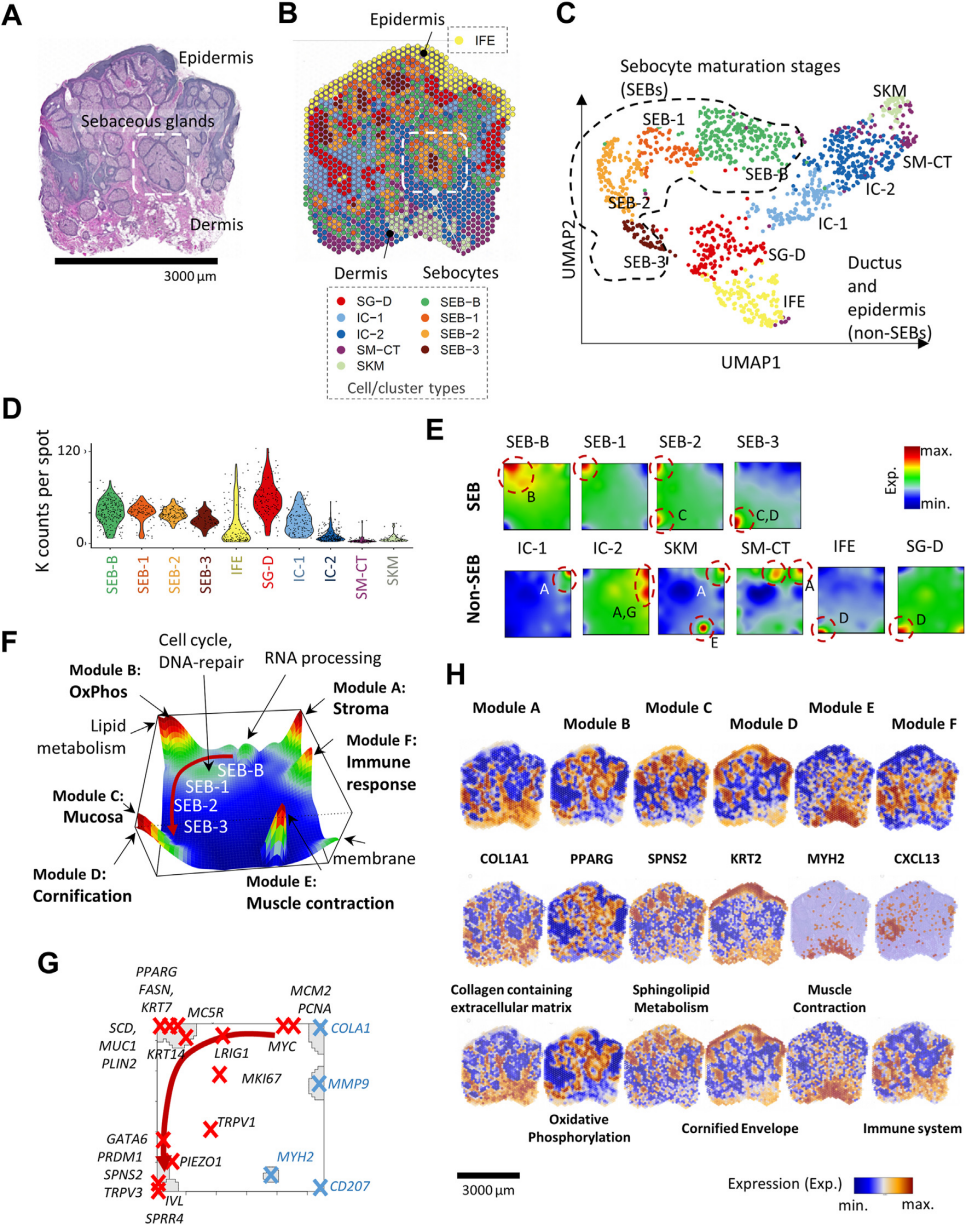
**Sebaceous gland hyperplasia spatial transcriptomics.***A*, H&E-stained and (*B*) spatial transcriptomics images. The colors of the spots refer to different cell types including sebocytes (SEB) and non-SEB types such as epidermal, ductal, immune, and muscle cells (see legend below the image). The box formed by *dashed lines* encloses an acinus formed by SEB clusters in a centripetal arrangement. *C*, UMAP of the spots distributes cell type-specific clusters along two major branches, one referring to development from SEB-B towards SEB-3, the other one to non-SEB cells, including sebaceous/hair follicle ductus (SG-D), interfollicular epidermis (IFE), connective tissue/smooth muscle (SM-CT), skeletal muscle (SK-M), and periadnexal (IC-1) as well as perivascular immune cells (IC-2). *D*, transcript counts for the spots per cluster. *E*, SOM portraits of the cell types characterize their typical expression patterns in terms of (*red*) overexpressed modules of co-expressed genes. *F*, assemblage of the modules in the SOM-expression landscape together with their functional context. The *red arrow marks* the developmental trajectory of SEBs. *G*, marker genes for different stages of SEB development distribute along this trajectory (*red crosses*). *H*, spatial images colored according to the expression of the modules, typical gene signatures, and selected individual genes.

Deriving valuable information from ST studies may be limited when analyzing small structures such as SGs. To overcome this hurdle, we analyzed sebaceous hyperplasia (Fig. 1*A*). Importantly, the relevant literature agrees that in sebaceous hyperplasia, the glandular acini are increased in size and number, but retain a normal structure (15, 16), For instance, in contrast to sebaceous adenoma, there is no increase in the number of germinative cells or other architectural abnormalities (17, 18). First, we visualized the sequencing data as ST image and in spots by Uniform Manifold Approximation and Projection (UMAP; Fig. 1, *B* and *C*, respectively). With an unsupervised approach based on gene expression similarities, combined with histological assessment, we identified ten clusters (Fig. 1, *C* and *D*): four comprising SG acinar cells (=sebocytes, SEB) and six of non-sebocyte origin, *i.e.*, sebaceous/hair follicle ducts (SG-D), interfollicular epidermis (IFE), connective tissue/smooth muscle (SM-CT), skeletal muscle (SK-M), as well as periadnexal (IC-1) and perivascular immune cells (IC-2). The highest transcript counts were detected for SEB and SG-D clusters (Fig. 1*D*; see Table S1 for cluster-specific gene expression data). Overlaying the cluster perimeters onto the H&E-stained images confirmed the distribution of SG cells in an onion skin-like concentric arrangement, with SEB-B forming the peripheral layer followed centripetally by SEB-1, SEB-2, and SEB-3 (white frame in Fig. 1*B*). The UMAP cluster plot (Fig. 1*C*) formed two distinct branches, one along different sebocyte types (SEB-B and SEB-1 to 3), and a second branch encompassing non-SEB cell types. Notably, the D-shaped arrangement of sebocyte clusters in the UMAP branches depicts their differentiation pathway and their proximity relationships to non-SEB cells. As spots may contain up to ten single cells, neighboring cells can be jointly captured, which may explain the adjacency of the SEB-B and SM-CT and of the SEB-3 and SG-D clusters in the UMAP and spatial images.

To better characterize the gene expression of the 11,181 genes measured in the spots, we utilized the oposSOM software for transcriptome analysis using Self-Organizing Maps (SOMs), a neural network machine learning approach (19). It reduces the dimension of the gene expression data and provides individual portraits of the transcriptome landscape of each spot. These portraits reveal specific modules of co-expressed genes (labeled A-F; enriched gene sets and statistical information are summarized in Tables S2 and S3), whose functional context can be assessed using gene set (GS) analysis. Each of the ten identified clusters represents a distinctive and unique transcriptional state, mainly characterized by the activation of one or two specific gene modules, as visualized by their average SOM portrait (Fig. 1*E*). SK-M, SM-CT, IC-1, and IC-2 portraits exhibited upregulation in the top right portrait area (associated with inflammation, extracellular matrix, and immune system genes in modules A and F, respectively), while SG-D and IFE displayed upregulation in the bottom area of the SOM (keratinization and epithelium in module D and partly in C). Interestingly, the development from SEB-B through SEB-1 and SEB-2 to SEB-3 is reflected by the activation of modules shifting from top-middle (cell cycle and RNA processing genes) to the top left (mitochondrial and oxphos functions and lipid metabolism, module B) towards the bottom left corner (modules C and D) in the counter-clockwise direction. This progression, indicated by the red arrow in Figure 1*F*, offers an overview of the modules upregulated in the individual cell types. SG-typical genes are shown in the gene map (Fig. 1*G*) where markers of sebocyte development (red crosses) are arranged along the trajectory in SOM-gene space (red arrow).

Next, we colored the spatial images according to the expression of genes taken from different modules. Thus, for instance, IC-1 and IC-2 spots are highlighted by spot A and SEB-1 and SEB2 by spot B (Fig. 1*H*, first row). Similar regions can be colored using functional signatures or single “marker” genes, thus associating spatial patterns with their functional context and individual markers (Fig. 1*G*, middle and lower rows). For example, in module B, the gene signature “oxidative phosphorylation” and the *PPARG* gene provide highly similarly-colored images highlighting SEB-1 and SEB-2 areas. Together with this publication, we provide an interactive web browser for inspecting the ST data under different aspects (http://gondwanaland.izbi.uni-leipzig.de:5978/?dataset=16, see Experimental procedures), particularly by coloring the image by selecting the expression of one of the about 11,000 genes measured and/or by one of more than 3000 gene signatures available in the browser’s repository.

### Pseudotime and RNA-velocity analyses confirm progress from SEB-B to SEB-3

**Figure 2:**
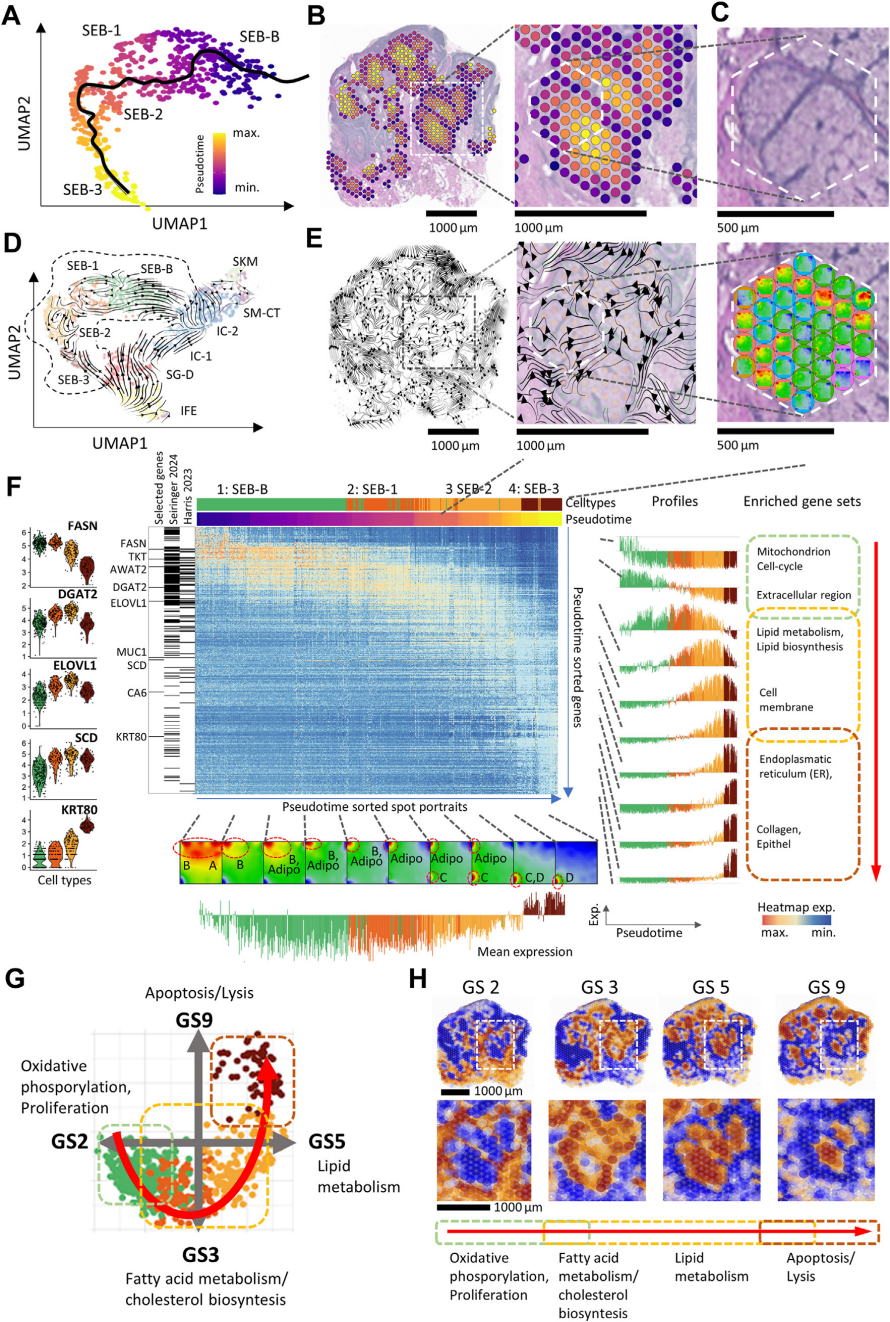
**Pseudotime dynamics of sebocyte development.***A*, pseudotime trajectory (*black curve*) through the sebocyte branch of the UMAP plot demonstrates cell development. *B*, pseudotime coloring of the image transfers developmental information into spatial coordinates. Note the concentric, centripetal developmental pattern. *C*, progressive pseudotime correlates with SEB-B, SEB-1, -2, and -3 cell types, as illustrated by the SOM portraits of the spots. *D* and *E*, RNA velocity in UMAP and in spatial coordinates suggest an “inside-out” direction of the vectors in the SG, reflecting loss of transcriptional activity of sebocytes with increasing pseudotime. *F*, Heatmap of gene expression along the pseudotime. The expression profiles (calculated as a mean value over 50 consecutive genes) at the *right* show how the expression maximum shifts along the pseudotime axis to the right. Selected genes are shown at the *left* together with their expression in the different cell types. The location of genes from independent transcriptomic studies along the PT are shown at the *left* as “barcode” (see also Fig. 3 and text). SOM portraits below the heatmap reflect changes in expression patterns with progressive sebocyte development. *G*, four gene set profiles (GS 2, 3, 5, 9) summarize the state space of sebocyte development as a continuum from SEB-B to SEB-3. *H*, the spatial images of the GS reflect the centripetal sebocyte development.

To further characterize sebaceous differentiation, we explored the development of SEB clusters using pseudotime (PT) dynamics. The PT trajectory mapped within the UMAP as a black curve, reflects a gradual and continuous differentiation from SEB-B to SEB-3 (Fig. 2*A*). Coloring the tissue image using the PT scale demonstrated progression from the SG periphery to the center, which reflects the spatial dynamics of sebocyte maturation (Fig. 2*B*). PT coloring reveals that our tissue slice contains about five larger and about five smaller individual glandular acini, all showing the PT-color gradient from dark blue to yellow. The PT trajectory in the UMAP (Fig. 2*A*) and the extracted PT genes (next subsection) thus refer to this collection of acinar structures and reflect a certain heterogeneity of the individual glandular acini in one ST sample. Zoom-in views show the respective areas in the H&E-stained image and as SOM portraits of the included spots (Fig. 2*C*, top and bottom parts, respectively), which reveal different module patterns agreeing with SEB-B to SEB-3 transcriptional landscapes (compared to Fig. 1*E*; analogous views of any region of the image can be obtained *via* the interactive spatial browser). Next, we calculated RNA-velocity vectors for the different spots in UMAP and spatial image coordinates using the software SIRV (20), a tool for inferring spatial differentiation trajectories (Fig. 2, *D* and *E*, respectively). RNA-velocity vectors point inside-out from central SEB-3 toward the surrounding SEB-B region (zoom in Fig. 2*E*), which indicates an imbalance between unspliced and spliced transcripts, thus predicting cellular state changes. The seemingly paradoxical direction of the vectors reflects the typical loss of transcriptional activity as sebocytes differentiate (see also Fig. 1*D* for counts distribution and mean expression plot in Fig. 2*F*). Importantly, both PT-trajectory and RNA-velocity analyses independently reveal progressive sebocyte maturation in spatial coordinates.

### Sebocyte differentiation reflects consecutive modulation of cellular metabolic functions

For a detailed view of the metabolic processes underlying sebocyte differentiation, we next generated a heatmap of gene expression along the PT trajectory (Fig. 2*F*). Marker genes for sebaceous development were retrieved from the literature and identified among pseudotime genes. The violin plots in the left part of Figure 2*F* reflect progressive gene activation with PT. More detailed PT profiles were obtained for sets of nearly 50 consecutive genes in the top-to-down list which robustly illustrate the left-to-right shift of maximum gene expression (Fig. 2*F*, right part). Thus, during the SEB-B to SEB-3 transition, we observed a shift from cell proliferation and oxphos activity (SEB-B) *via* lipid metabolism driving sebum production (SEB-1 and -2) towards lysis and apoptosis-related functions (SEB-3). A row of SOM portraits below the heatmap illustrates the transcriptional landscape changes along the PT: red overexpression modules shift from modules A and B for SEB-B and SEB-1 toward modules C and D for SEB-3 as illustrated in Figure 1*F* by the red dashed arrow. Interestingly, SEB-2 reflects a bimodal transition state with upregulated modules B and D. The upregulation of modules C and D in SEB-3 may be attributed to SG-D cells intermixing with sebocytes near the ductus where sebum leaves the SG. By employing four selected GS we constructed a two-dimensional state space that supports a continuous sebocyte development by consecutive upregulation of cellular functions associating with the GS modules (Fig. 2*G*) which refer to modules A-D in the portraits (not shown). The expression of the modules indicates the activated regions in the spatial image (Fig. 2*H*).

### Validation using independent sebaceous transcriptome data and immunohistochemistry

The employed sebaceous hyperplasia tissue sample is characterized by glandular acini having a normal structure, only being increased in size and number. However, it cannot be fully excluded that the cellular transcriptional program or metabolic pathways are altered due to the hyperplastic phenotype. Thus, to confirm the biological relevance of our data, we next evaluated the expression of the proteins corresponding to transcripts with a high fold-change in each SEB cluster (see Table S1) by accessing the Human Protein Atlas (21). The proteins assessed for SEB-B were transketolase (TKT), a key enzyme of the pentose phosphate pathway (22), and heat shock protein family D member 1 (HSPD1), a chaperonin important for mitochondrial function (23), which together reflect the intense oxidative/metabolic activity of sebocytes at this stage. The SEB-1 stage was defined by acyl-CoA wax alcohol acyltransferase 2 (AWAT2), involved in the synthesis of sebum wax esters (24), and endogenous retrovirus group 3 member 1 (ERV3-1), an endogenous retroviral element of the human genome known to be expressed in sebocytes but with no identified functions in SGs (25). SEB-2 was defined by ethylmalonyl-CoA decarboxylase (ECHDC1), an essential enzyme for accurate FA synthesis (26), and glutaredoxin-1 (GLRX), involved in redox regulation and previously demonstrated to be important for proper hepatic lipogenesis (27). Finally, SEB-3 sebocytes were defined by corneodesmosin (CSDN), an adhesive protein essential for terminal keratinocyte differentiation, whose expression and function in SGs has not been appreciated so far (28), as well as gasdermin-A (GSDMA), pore-forming protein with important functions in cell death and immunity (29).

**Figure 3:**
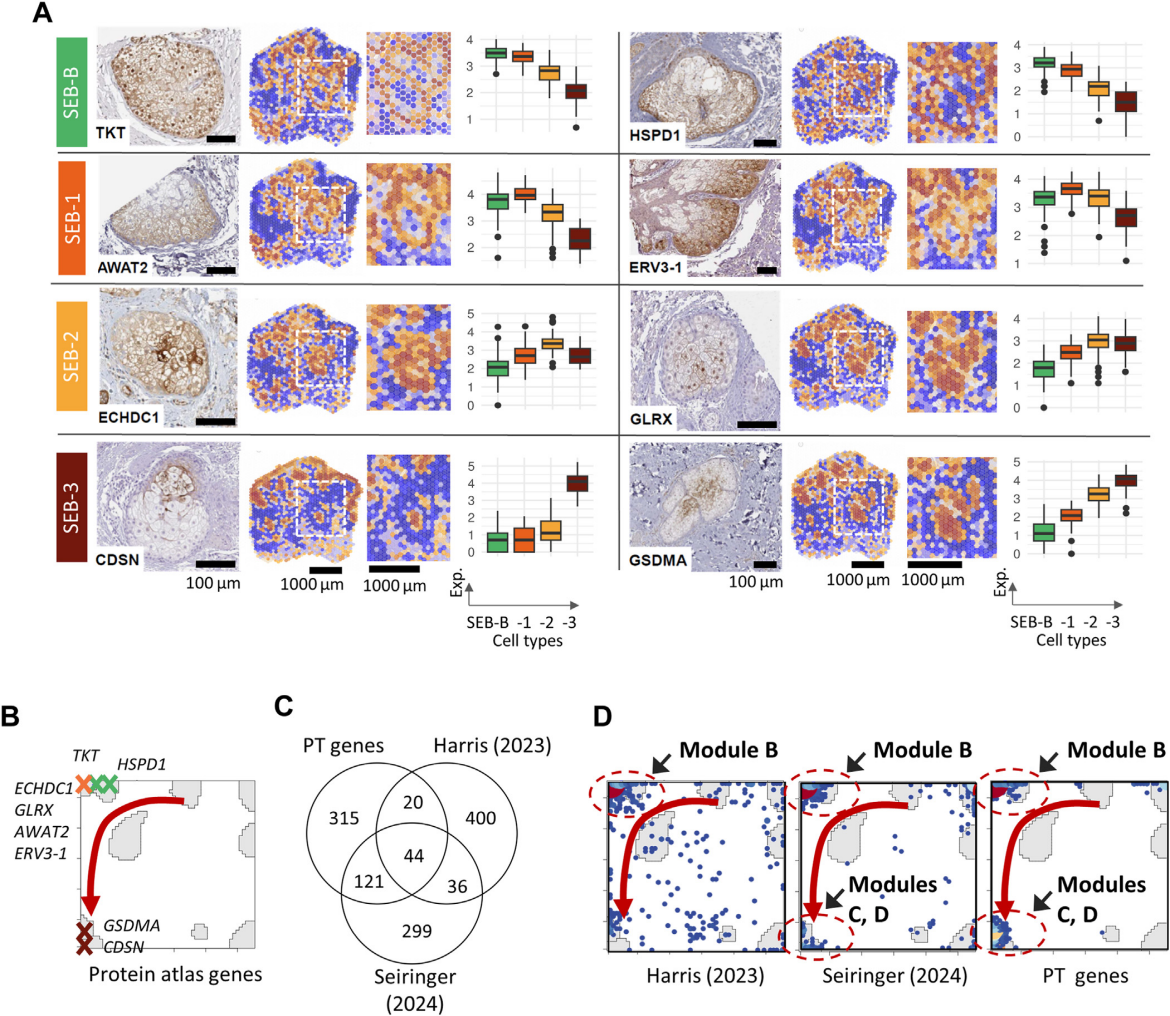
**Validation using independent proteomics and transcriptomics data.***A*, immunohistochemically-stained images of human SGs obtained from the Human Protein Atlas (https://www.proteinatlas.org/) (21), ST images of the coding genes, and boxplots of the expression across the SG cell types (from *left* to the *right*). The proteins/transcripts selected from the four groups SEB-B, -1, -2 and -3 were transketolase (TKT), heat shock protein family D member 1 (HSPD1), acyl-CoA wax alcohol acyltransferase 2 (AWAT2), endogenous retrovirus group 3 member 1 (ERV3-1), ethylmalonyl-CoA decarboxylase (ECHDC1), glutaredoxin-1 (GLRX), corneodesmosin (CSDN), and gasdermin-A (GSDMA). Protein and transcript-stained images mark similar SG-regions. *B*, the selected genes group along the SG-developmental trajectory in the SOM (compare with Fig. 1*G*). *C*, Venn diagram of the top 500 differentially expressed SG-specific genes (DEG) taken from Seiringer *et al.* (14) and Harris *et al.* (13) and from our study (PT-genes) and (*D*) of their gene maps in the SOM. The genes group along the PT-trajectory (*red arrow*) where the DEG from independent studies accumulate slightly in the areas referring to SEB-B and -1 (see also the “barcode”-genes in Fig. 2*F*).

SGs are not specifically annotated in this resource, but screening immunohistochemically-stained skin samples allowed to establish an excellent correlation between individual SEB cluster-specific transcripts and the spatial localization of the corresponding protein in the SG *in situ* (Fig. 3*A*; compare the enlarged ST-images and boxplots with the respective protein-stained SG-sections). Thus, expression of TKT and HSPD1 (both SEB-B) was highest in peripheral sebocytes, of AWAT2, ERV3-1, ECHDC1, and GLRX (SEB-1 and 2) in middle-SG sebocytes, and of CDSN and GSDMA (SEB-3) in centrally located SG cells. These genes also group along the SG-developmental trajectory in the SOM expression landscape (red arrow in Fig. 3*B*). Altogether, comparison of the protein-stained images with their respective gene expression-colored ST images and SEB stage affiliation shows remarkably similar, centripetally developing patterns for the SEB-B, -1, 2, and 3 groups.

For a second line of verification analysis, we compared specific differentially expressed SG-genes (DEG) taken from recent studies of Seiringer *et al.* (14) and Harris *et al.* (13) with our genes identified in the PT analysis (Fig. 2*F*). The Venn diagram in Figure 3*C* shows good overlap between the top 500 PT and the two independent DEG lists. We have previously shown that overlap between independent marker sets is limited by intrinsic errors of the data and the number of coregulated genes even if one compares identical phenotypes (30). In our modules B-D we identified more than 1159 co-regulated genes, which associate with SEB differentiation. Hence, the agreement between our spatial and the used single-cell data suggests virtually unique mechanisms of sebocyte maturation given the data presently available. More subtle differences cannot be excluded but will need larger data.

Overlap genes of the independent studies of Harris *et al.* and Seiringer *et al.* both accumulate at smaller PT-values in earlier developmental stages SEB-B, -1 and partly SEB-2 (see “barcode” at the left side of the heatmap in Fig. 2*F*) owing to the fact that DEG-analysis is biased towards the highly variant genes found at low PT-values. In addition, as DEGs are calculated contrasting against non-SG cells, SEB-3 to some extent overlaps with a fraction of non-SEB ductal cells (see above), which results in a partial depletion of DEGs in SEB-3. Despite this biased accumulation, the DEGs from both independent studies groups along the PT-trajectory in the SOM, particularly in and near the modules B (SEB-B, -1), C, and D (SEB-2, -3, see red arrows in Fig. 3*D*). Thus, a comparison of our ST results with recent independent transcriptomics studies and with protein-staining in SG tissue sections clearly confirms the developmental dynamics of the SG groups identified in this study.

In summary, by integrating single-cell spatial transcriptomics, pseudotime analysis, RNA velocity, and functional enrichment, we provide a finely granulated and comprehensive understanding of the transcriptional dynamics underlying sebaceous differentiation. The data support continuous sebocyte development by consecutive modulation of cellular functions that can be associated with specific gene sets. By delivering novel candidates to modulate SG function in skin diseases, our study delineates future experimental approaches of translational relevance.

## Experimental procedures

### Tissue sample processing

The sebaceous hyperplasia sample employed in this study was collected with ethical approval by the Dessau University Hospital and after patient consent for use of the specimen for research purposes, and in full compliance to the Declaration of Helsinki principles. The sample was formalin-fixed, paraffin-embedded, and spatial transcriptomic analysis was carried out with the Visium platform (10× Genomics) according to the manufacturer’s instructions by a qualified service provider (Indivumed Services). Sequencing data were processed using the Space Ranger software (10× Genomics). Quality control metrics, including library saturation and feature-spot distribution assessment, were evaluated and found within the recommended range.

### Bioinformatic analysis

Alignment to the human genome (GRCh38) and quantification of gene expression were conducted. Spots were annotated based on their coordinates on the tissue section. Downstream analyses were conducted using R v4.3.2 and Python v3.8.5, with processed data to be made available on request. The analysis was initiated with a comprehensive approach to dimensionality reduction and visualization using the Seurat R package (version 4.3.0.1 (31)). Data preprocessing was performed to filter out low-quality cells and normalize gene expression levels. Principal component analysis (PCA) was then executed to identify the most significant components, thereby capturing the variance and underlying structure of the data. Subsequently, the UMAP technique was applied, utilizing its default parameters for visualizing the high-dimensional data in a two-dimensional space. For cell categorization based on gene expression profiles, we employed the shared nearest neighbor (SNN) modularity optimization-based clustering algorithm. Each identified cluster was further assigned a specific functional skin cell type. This assignment was based on the identification of differentially expressed genes, achieved through statistical tests comparing gene expression between clusters. The results of this differential expression analysis are summarized in Table S1. The identified clusters, characterized by their distinct cell types, were then overlaid on corresponding Hematoxylin and Eosin (H&E) stained images and UMAP plots. This overlay process enabled the correlation of spatial and transcriptomic data, providing a comprehensive view of the spatial distribution and molecular characteristics of the various skin cell types within the clusters.

The pseudotemporal dynamics of sebocyte differentiation were explored using Monocle 3 (version 1.0.0 (32)), which enabled the visualization of the continuous differentiation process. It first fits a principal graph within the sebocyte partition in the UMAP. After defining the cluster SEB-B as the “beginning” of the biological process of differentiation, the monocle ordered the cells according to their progress through development. Subsequent genes showing differential expression along the pseudotime trajectory were identified. These genes were crucial in understanding the dynamic changes occurring during sebocyte maturation.

After this identification, we conducted a functional enrichment analysis of the differentially expressed genes using the DAVID Bioinformatics Resources. This step was crucial in revealing the underlying biological processes, pathways, and functional categories associated with the genes along the pseudotime trajectory. Additionally, the average expression of metagene modules, derived from this analysis, was mapped onto spatial images. This mapping provided a spatial context to the gene expression changes observed during sebocyte differentiation, integrating transcriptomic data with physical tissue structure. Spatial differentiation trajectories within the spatial context of tissue were inferred using RNA velocity, calculated by the software SIRV (https://github.com/tabdelaal/SIRV/tree/main, (20)). This analysis provided insights into the directional differentiation of sebocytes within the gland.

High-dimensional expression data of 11,181 gene transcripts were transformed into 3025 metagenes using the oposSOM software (version 2.2.8 (19)), and these metagenes were arranged in a 55 × 55 grid. Genes with similar expression profiles were grouped together, resulting in the visualization of each cell's transcriptome landscape, termed its ‘SOM portrait’, where overexpressed and underexpressed metagenes were color-coded in red and blue, respectively. Spot-like areas of red and blue, indicative of over- and under-expression, emerged on the grid due to the self-organizing properties of the SOM, forming clusters of mutually correlated genes. These modules were detected based on an overexpression criterion, utilizing metagene expression as previously described (33).

The expression patterns of these modules serve as a unique characteristic fingerprint for each sample, capturing the intricate details of gene expression interplay. Lists of genes within each module, along with lists of enriched gene sets and statistical information, are summarized in Tables S2 and S3. An interactive web-based tool (http://gondwanaland.izbi.uni-leipzig.de:5978/?dataset=16) was developed in the frame of the oposSOM browser (19), allowing exploration of the spatial transcriptomics data in various dimensions, including gene expression, gene signatures, and functional annotations (34).

### Assessment of protein expression in SG tissue sections

The spatial localization of proteins corresponding to transcripts marking the different sebocyte differentiation stages was assessed by examining immunohistochemically-stained tissue sections from the Human Protein Atlas (21). For SEB-B: TKT (https://www.proteinatlas.org/ENSG00000163931-TKT/tissue/skin) and HSPD1 (https://www.proteinatlas.org/ENSG00000144381-HSPD1/tissue/skin); For SEB-1: AWAT2 (https://www.proteinatlas.org/ENSG00000147160-AWAT2/tissue/skin) and ERV3-1 (https://www.proteinatlas.org/ENSG00000213462-ERV3-1/tissue/skin); For SEB-2: ECHDC1 (https://www.proteinatlas.org/ENSG00000093144-ECHDC1/tissue/skin) and GLRX (https://www.proteinatlas.org/ENSG00000173221-GLRX/tissue/skin); For SEB-3: CDSN (https://www.proteinatlas.org/ENSG00000204539-CDSN/tissue/skin) and GSDMA (https://www.proteinatlas.org/ENSG00000167914-GSDMA/tissue/skin). The specific information on staining methods and employed antibodies can be found by consulting the repository at (https://www.proteinatlas.org/).

## Data availability

Datasets related to this article can be found at the public Leipzig Health Atlas repository (https://www.health-atlas.de/) under the accession number LHA ID: 8MWXC02K01-6.

## Supporting information

This article contains supporting information.

## Conflict of interest

The authors declare that they have no known competing financial interests or personal relationships that could have appeared to influence the work reported in this paper.

## References

[1] Schneider M.R., Paus R. (2010). Sebocytes, multifaceted epithelial cells: lipid production and holocrine secretion. Int. J. Biochem. Cell Biol..

[2] Hinde E., Haslam I.S., Schneider M.R., Langan E.A., Kloepper J.E., Schramm C. (2013). A practical guide for the study of human and murine sebaceous glands in situ. Exp. Dermatol..

[3] Dahlhoff M., Camera E., Schafer M., Emrich D., Riethmacher D., Foster A. (2016). Sebaceous lipids are essential for water repulsion, protection against UVB-induced apoptosis and ocular integrity in mice. Development.

[4] Kobayashi T., Voisin B., Kim D.Y., Kennedy E.A., Jo J.-H., Shih H.-Y. (2019). Homeostatic control of sebaceous glands by innate lymphoid cells regulates commensal bacteria equilibrium. Cell.

[5] Choa R., Tohyama J., Wada S., Meng H., Hu J., Okumura M. (2021). Thymic stromal lymphopoietin induces adipose loss through sebum hypersecretion. Science.

[6] Williams H.C., Dellavalle R.P., Garner S. (2012). Acne vulgaris. Lancet.

[7] Zouboulis C.C., Picardo M., Ju Q., Kurokawa I., Torocsik D., Biro T. (2016). Beyond acne: current aspects of sebaceous gland biology and function. Rev. Endocr. Metab. Disord..

[8] Negri V.A., Watt F.M. (2022). Understanding human epidermal stem cells at single-cell resolution. J. Invest. Dermatol..

[9] Jin S., Ramos R. (2022). Computational exploration of cellular communication in skin from emerging single-cell and spatial transcriptomic data. Biochem. Soc. Trans..

[10] Veniaminova N.A., Jia Y.Y., Hartigan A.M., Huyge T.J., Tsai S.-Y., Grachtchouk M. (2023). Distinct mechanisms for sebaceous gland self-renewal and regeneration provide durability in response to injury. Cell Rep..

[11] Thalheim T., Schneider M.R. (2024). Skin single-cell transcriptomics reveals a core of sebaceous gland-relevant genes shared by mice and humans. BMC Genomics.

[12] Rao A., Barkley D., França G.S., Yanai I. (2021). Exploring tissue architecture using spatial transcriptomics. Nature.

[13] Harris J.C., Prouty S.M., Nelson M.A., Sung D.C., Nelson A.M., Seykora J.T. (2023). Laser capture microdissection-based RNAseq for profiling mouse and human sebaceous gland transcriptomes. J. Invest. Dermatol..

[14] Seiringer P., Hillig C., Schäbitz A., Jargosch M., Pilz A.C., Eyerich S. (2024). Spatial transcriptomics reveals altered lipid metabolism and inflammation-related gene expression of sebaceous glands in psoriasis and atopic dermatitis. Front. Immunol..

[15] Iacobelli J., Harvey N.T., Wood B.A. (2017). Sebaceous lesions of the skin. Pathology.

[16] Eisen D.B., Michael D.J. (2009). Sebaceous lesions and their associated syndromes: part I. J. Am. Acad. Dermatol..

[17] Kazarov D.V., Michal M., Kacerovska D., McKee P.H. (2012).

[18] Requena L., Sangueza O. (2017).

[19] Löffler-Wirth H., Kalcher M., Binder H. (2015). oposSOM: R-package for high-dimensional portraying of genome-wide expression landscapes on bioconductor. Bioinformatics.

[20] Abdelaal T., Lelieveldt B.P., Reinders M.J., Mahfouz A. (2021). SIRV: spatial inference of RNA velocity at the single-cell resolution. bioRxiv.

[21] Uhlen M., Fagerberg L., Hallstrom B.M., Lindskog C., Oksvold P., Mardinoglu A. (2015). Proteomics. Tissue-based map of the human proteome. Science.

[22] Horecker B.L. (2002). The pentose phosphate pathway. J. Biol. Chem..

[23] Viitanen P.V., Lorimer G.H., Seetharam R., Gupta R.S., Oppenheim J., Thomas J.O. (1992). Mammalian mitochondrial chaperonin 60 functions as a single toroidal ring. J. Biol. Chem..

[24] Turkish A.R., Henneberry A.L., Cromley D., Padamsee M., Oelkers P., Bazzi H. (2005). Identification of two novel human acyl-CoA wax alcohol acyltransferases: members of the diacylglycerol acyltransferase 2 (DGAT2) gene superfamily. J. Biol. Chem..

[25] Andersson A.C., Merza M., Venables P., Pontén F., Sundström J., Cohen M. (1996). Elevated levels of the endogenous retrovirus ERV3 in human sebaceous glands. J. Invest. Dermatol..

[26] Dewulf J.P., Paquay S., Marbaix E., Achouri Y., van Schaftingen E., Bommer G.T. (2021). ECHDC1 knockout mice accumulate ethyl-branched lipids and excrete abnormal intermediates of branched-chaifatty acid metabolism. J. Biol. Chem..

[27] Di Shao, Han J., Hou X., Fry J., Behring J.B., Seta F. (2017). Glutaredoxin-1 deficiency causes fatty liver and dyslipidemia by inhibiting sirtuin-1. Antioxid. Redox Signal..

[28] Leclerc E.A., Huchenq A., Mattiuzzo N.R., Metzger D., Chambon P., Ghyselinck N.B. (2009). Corneodesmosin gene ablation induces lethal skin-barrier disruption and hair-follicle degeneration related to desmosome dysfunction. J. Cell Sci..

[29] Ding J., Wang K., Liu W., She Y., Sun Q., Shi J. (2016). Pore-forming activity and structural autoinhibition of the gasdermin family. Nature.

[30] Loeffler-Wirth H., Kreuz M., Schmidt M., Ott G., Siebert R., Binder H. (2022). Classifying germinal center derived lymphomas-navigate a complex transcriptional landscape. Cancers (Basel).

[31] Hao Y., Hao S., Andersen-Nissen E., Mauck W.M., Zheng S., Butler A. (2021). Integrated analysis of multimodal single-cell data. Cell.

[32] Cao J., Spielmann M., Qiu X., Huang X., Ibrahim D.M., Hill A.J. (2019). The single-cell transcriptional landscape of mammalian organogenesis. Nature.

[33] Wirth H., Löffler M., Bergen M.V., Binder H. (2011). Expression cartography of human tissues using self organizing maps. BMC Bioinformatics.

[34] Schmidt M., Avagyan S., Reiche K., Binder H., Loeffler-Wirth H. (2024). A spatial transcriptomics browser for discovering gene expression landscapes across microscopic tissue sections. Curr. Issues Mol. Biol..

